# Supplementary figures and images for: Bazedoxifene does not share estrogens effects on IgG sialylation
Source: PLoS One. 2023 May 18;18(5):e0285755. doi: 10.1371/journal.pone.0285755 (PMC10194887; doi:10.1371/journal.pone.0285755)

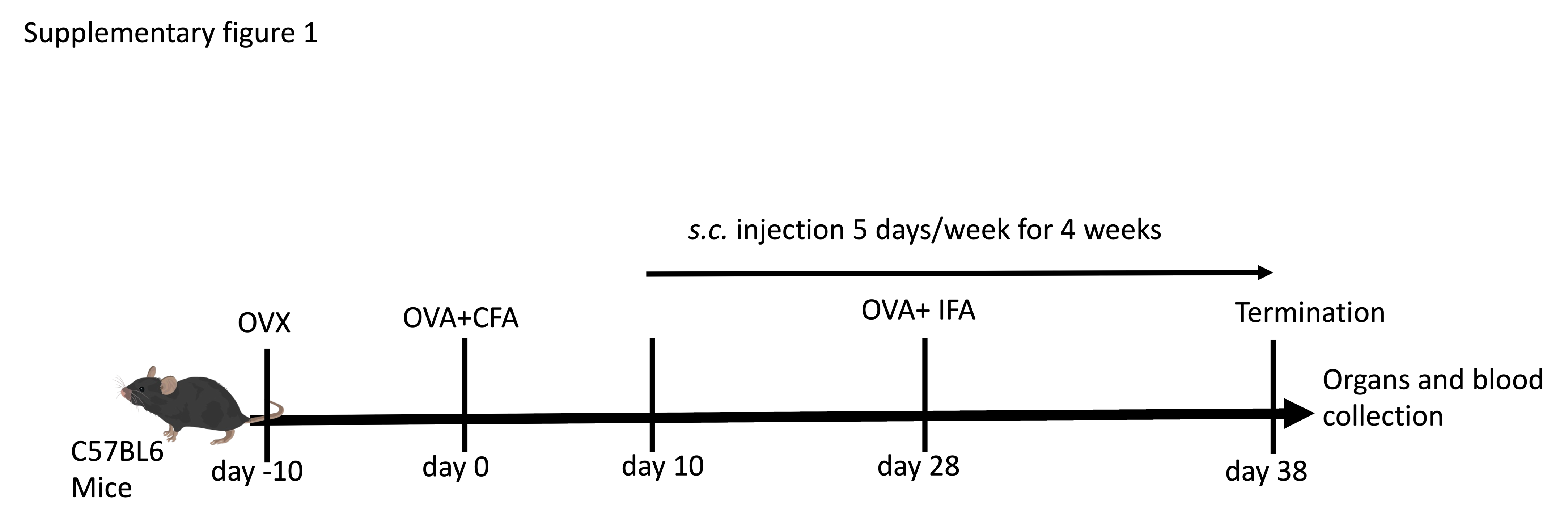

Supplement: S1 Fig — Female C57Bl6 mice were ovariectomized at 9 weeks of age (day -10). On day 0 mice were 1st immunized with OVA and complete Freund’s adjuvants (CFA) subcutaneously. On day 10, treatment was initiated with hormones. Mice received 2nd immunization with OVA and incomplete Freund’s adjuvant (IFA) on day 28. Mice were terminated on day 38. (TIFF) [file pone.0285755.s001.tiff]

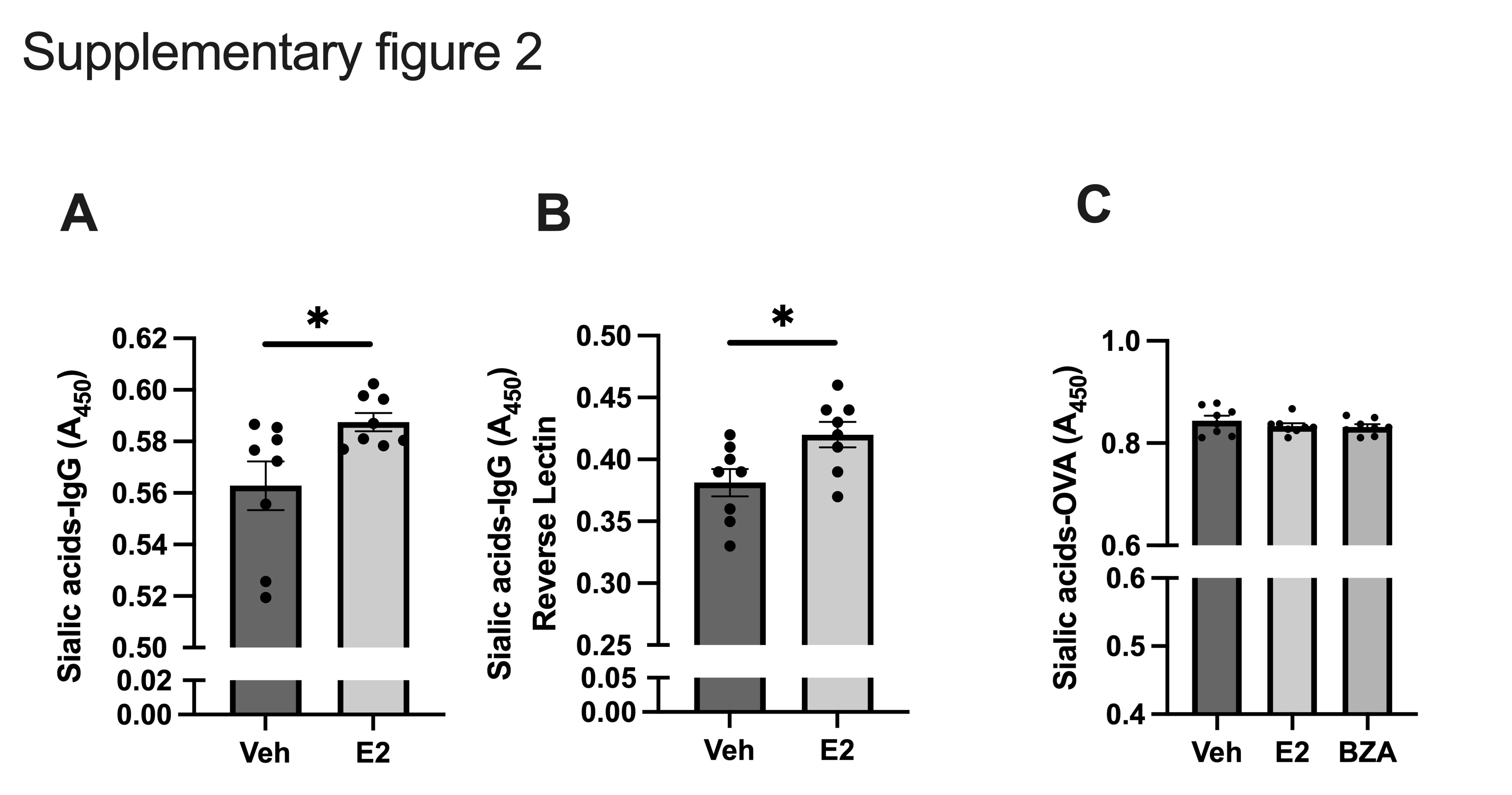

Supplement: S2 Fig — (A) the concentration of sialic acid on total IgG (lectin ELISA), (B) the concentration of sialic acid on total IgG (reverse lectin ELISA) Student’s t-test used to assess the difference. (C) Sialic acid on OVA-IgG. One-way ANOVA followed by Dunnett’s multiple comparisons to assess the difference towards a vehicle. *P < 0.05, A (450): Absorbance (450 nm). (n = 8/group). E2: 17β-estradiol-3-benzoate, BZA: Bazedoxifene, veh: Vehicle. (TIFF) [file pone.0285755.s002.tiff]

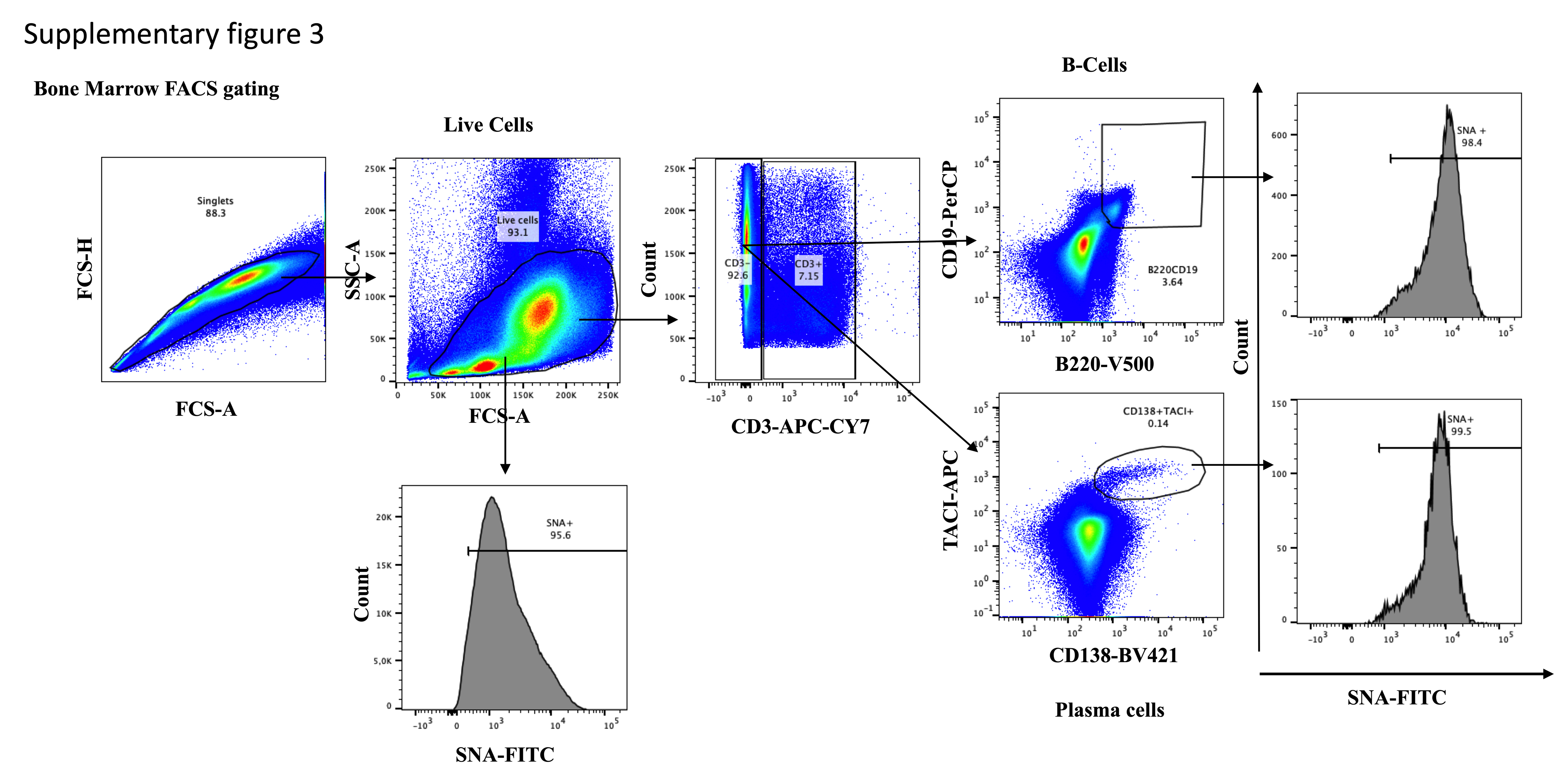

Supplement: S3 Fig — Singlets were determined using FSC-Height versus FSC-Area followed by live cell gating based on size and granularity. CD3− cells were gated into B- cells (double positive of CD19+ and B220+), and plasma cells (CD138+ TACI+). In live, B and plasma cells sialic acids were detected intracellular using mean fluorescence intensity (MFI) of sambuca nigra lectin (SNA). (TIFF) [file pone.0285755.s003.tiff]
