# Supplementary material for: Bazedoxifene does not share estrogens effects on IgG sialylation
Source: PLoS One. 2023 May 18;18(5):e0285755. doi: 10.1371/journal.pone.0285755 (PMC10194887; doi:10.1371/journal.pone.0285755)
Supplement: Attachment — Submitted filename: New Microsoft Word Document.docx [file pone.0285755.s004.docx]

The paper entitled “Bazedoxifene does not share estrogens effects on IgG sialylation” investigated effects of bazedoxifene and E2 on the IgG sialylation. However, several issues should be addressed:

1- The initial description of the abstract is not complete and coherent.
2- This study requires a control group. Why didn't you study a control group in addition to the Vehicle group?
3- How was the sample size calculated?
4- How many mice were in each group?
5- In result section, you said "Neither of the treatment affects the mRNA levels of glycosyltransferases in ovariectomized OVA immunized mice" but in figure 3 there is significance difference. Also the text of lines 188-192, does not coordinate with the diagrams A and B (about significant signs of the figure 3).
6- In discussion section this sentence need references: "Consistent with previous studies, we could not find any alteration of sialic acid in total serum protein by any of the treatments, suggesting that sialic acid levels in serum protein are independent of estrogen status."
